# Supplementary material for: Targeting Ultrasmall Gold Nanoparticles with cRGD Peptide Increases the Uptake and Efficacy of Cytotoxic Payload
Source: Nanomaterials (Basel). 2022 Nov 15;12(22):4013. doi: 10.3390/nano12224013 (PMC9696180; doi:10.3390/nano12224013)
Supplement: Supplementary file 1 [file nanomaterials-12-04013-s001.zip › nanomaterials-2010014-supplementary.pdf]

Targeted ultrasmall gold nanoparticles increase the uptake and efficacy of cytotoxic payload

Richard D. Perrins [1†], Lee-Anne McCarthy [2†], Angela Robinson [1†], Kelly L. Spry [1], Valentin Cognet [1], Avelino Ferreira [1,3], John Porter [1], Cristina Espinosa García [1], Miguel Ángel Rodriguez [1], Diana Lopez [1], Ibon Perera [1], Kelly Conlon [1], Africa Barrientos [1], Tom Coulter [1], Alessandro Pace [1], Sarah J. M. Hale [1], Enrico Ferrari [2], Csanad Z. Bachrati [2*]

1 Midatech Pharma Plc, 1 Caspian Point, Caspian Way, Cardiff CF10 4DQ, United Kingdom
2 School of Life Sciences, University of Lincoln, Joseph Banks Laboratories, Green Lane, Lincoln, LN6 7DL, United Kingdom
3 Present address: Department of Chemistry, Royal College of Surgeons in Ireland (RCSI), Dublin 2, Ireland
† Richard D. Perrins, Lee-Anne McCarthy and Angela Robinson contributed equally to this work.
* Correspondence: Email: cbachrati@lincoln.ac.uk; Tel.: +44 1522 886787

Supplementary Material

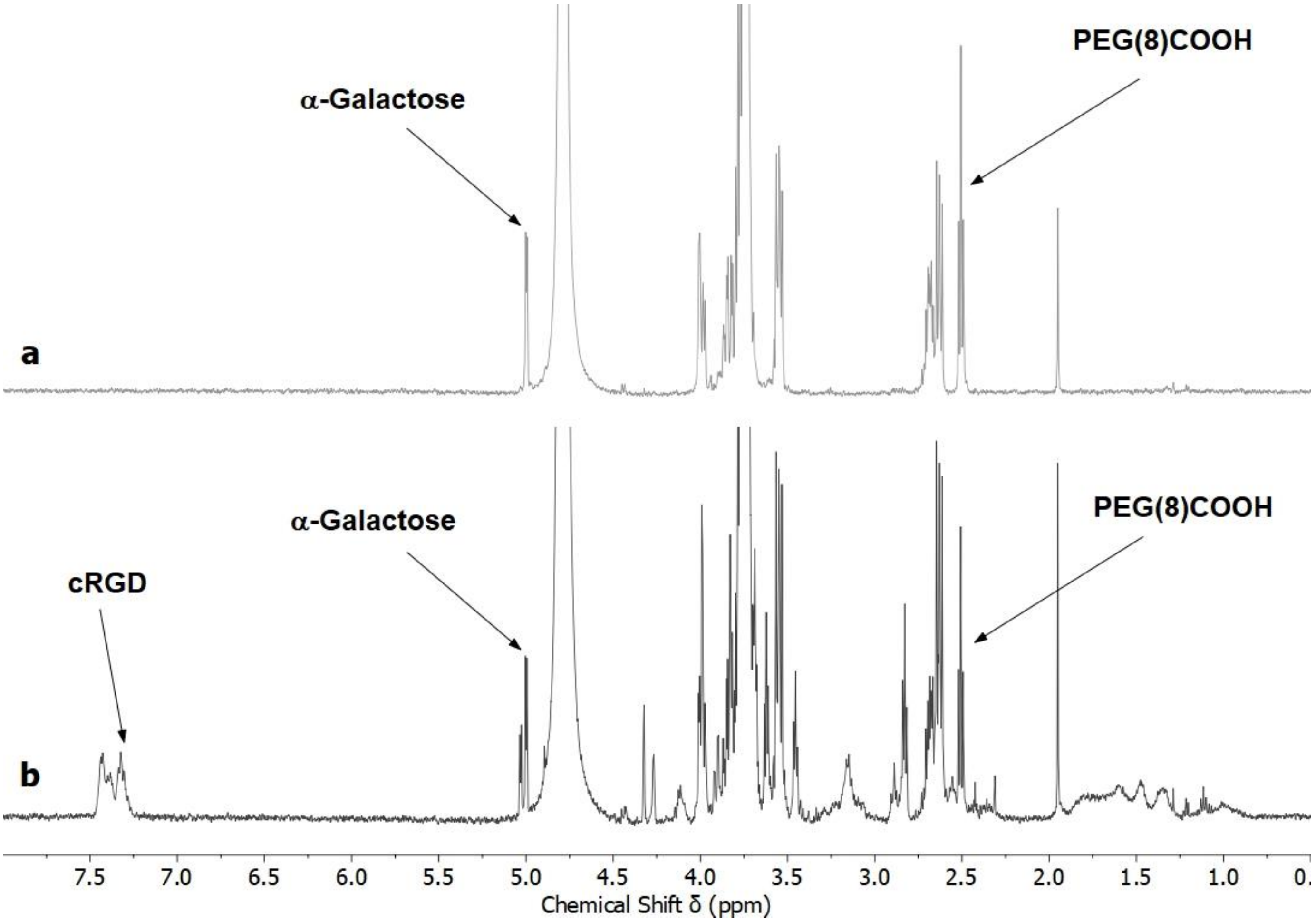

Figure S1. ^{1}H NMR (500 MHz, D_2O, δ) of (**a**) Base usGNP and (**b**) cRGD-usGNP. usGNPs were treated using a solution of potassium cyanide in potassium hydroxide (KCN/KOH) to etch the core and release the ligands. Signals belonging to the different ligands were chosen for their resolution and their areas were used to determine the relative amounts (area divided by the number of protons integrated). α-Galactose-C_2 5.00 (d, 1H, H-1). PEG(8)COOH 2.51 (t, 2H, CH_2-COOH). cRGD 7.38 (m, 5H, Ph). cRAD (not shown) 7.31 (m, 5H, Ph).

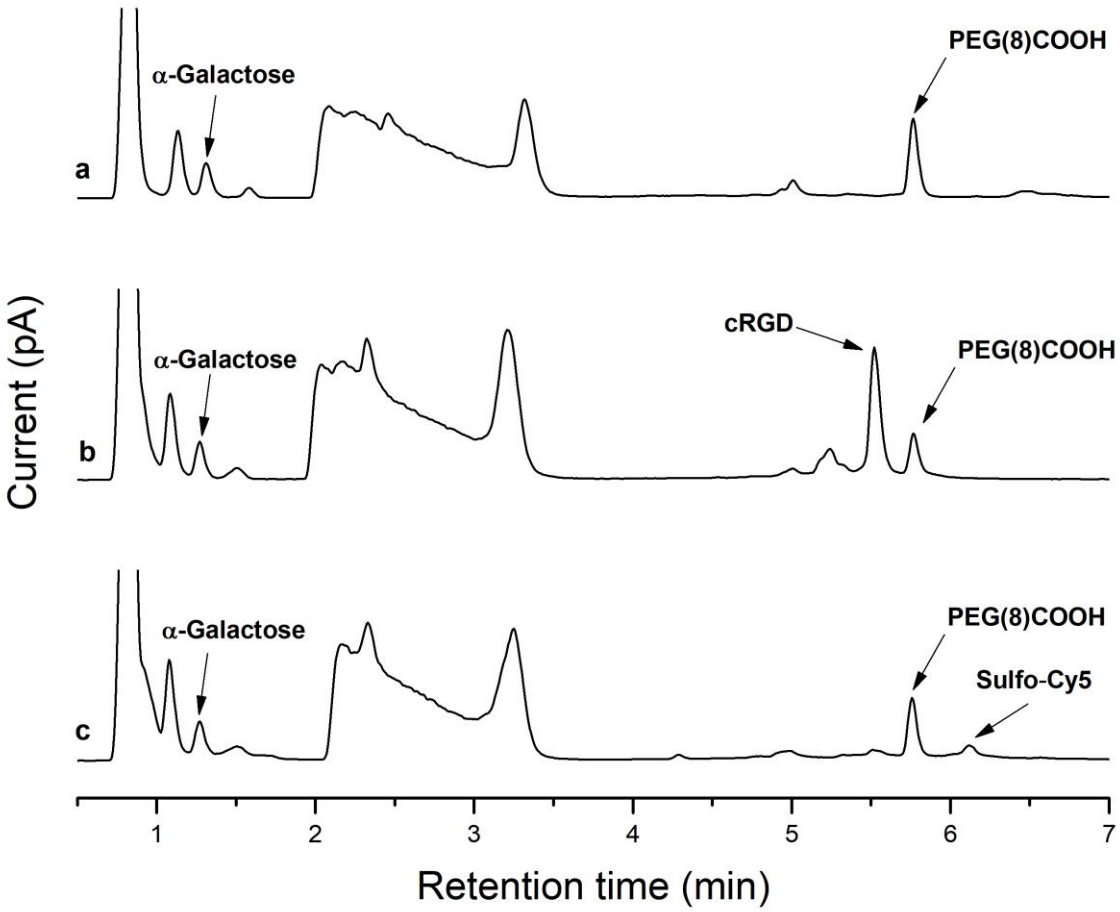

Figure S2. LC-CAD chromatogram with MS assignation of (**a**) Base GNP, (**b**) cRGD-GNP and (**c**) Cy5-GNP. usGNPs were treated with KCN/KOH and TCEP to release the ligands and maintain them as thiols. The retention times (RT) and the molecular weights (m/z) permitted identification of the ligands. The areas obtained with the CAD detector provided the relative amounts of ligands in the corona (area divided by the molecular weight of the ligand). α-Galactose-C_2 (RT: 1.271 min, m/z: N/A), cRGD derivative (RT: 5.522, m/z: 1334), cRAD derivative (not shown) (RT: 5.531, m/z: 1348), PEG(8)COOH (RT: 5.758 min, m/z: 459, 476) and Sulfo-Cyanine5 amine derivative (RT: 6.120, m/z fragmented: 894, 911).

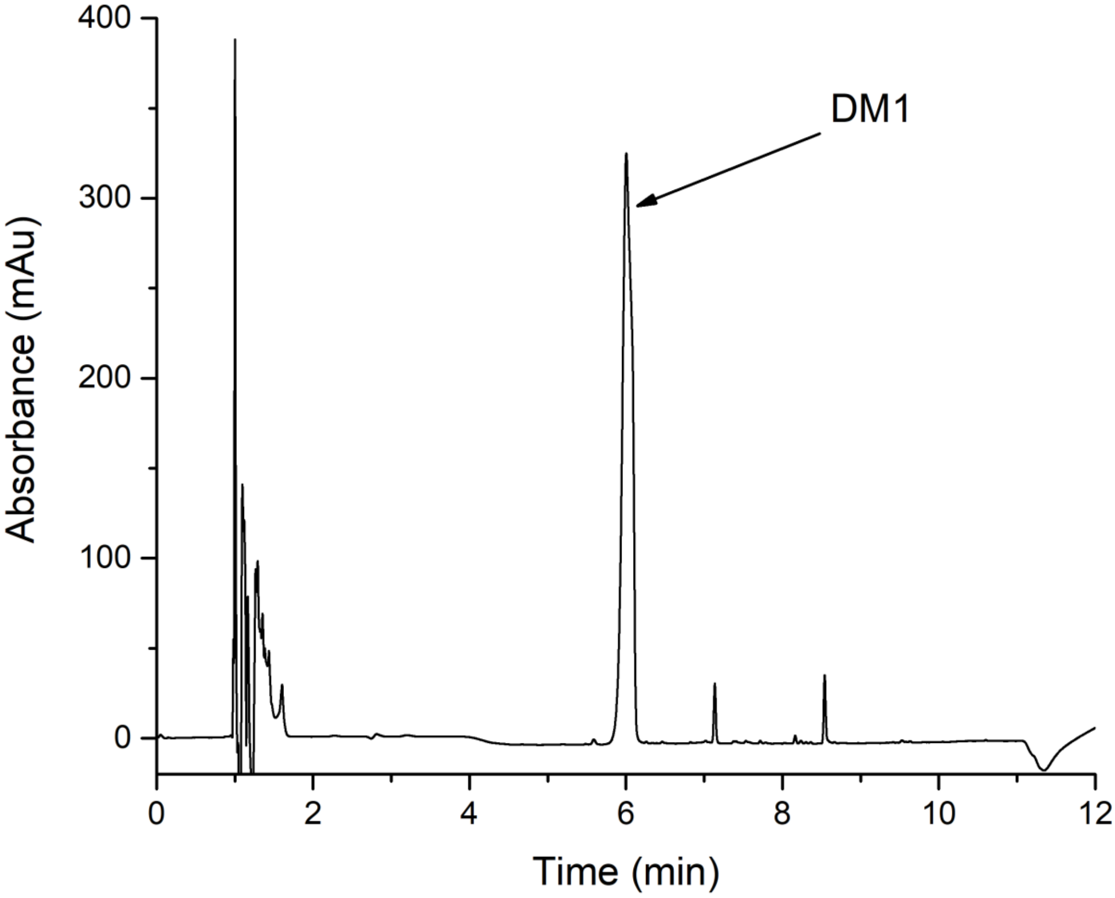

Figure S3. HPLC-UV chromatograms (λ 276 nm) of DM1-usGNP after particle etching and ligand release. DM1 peak elutes at 6 min.

a

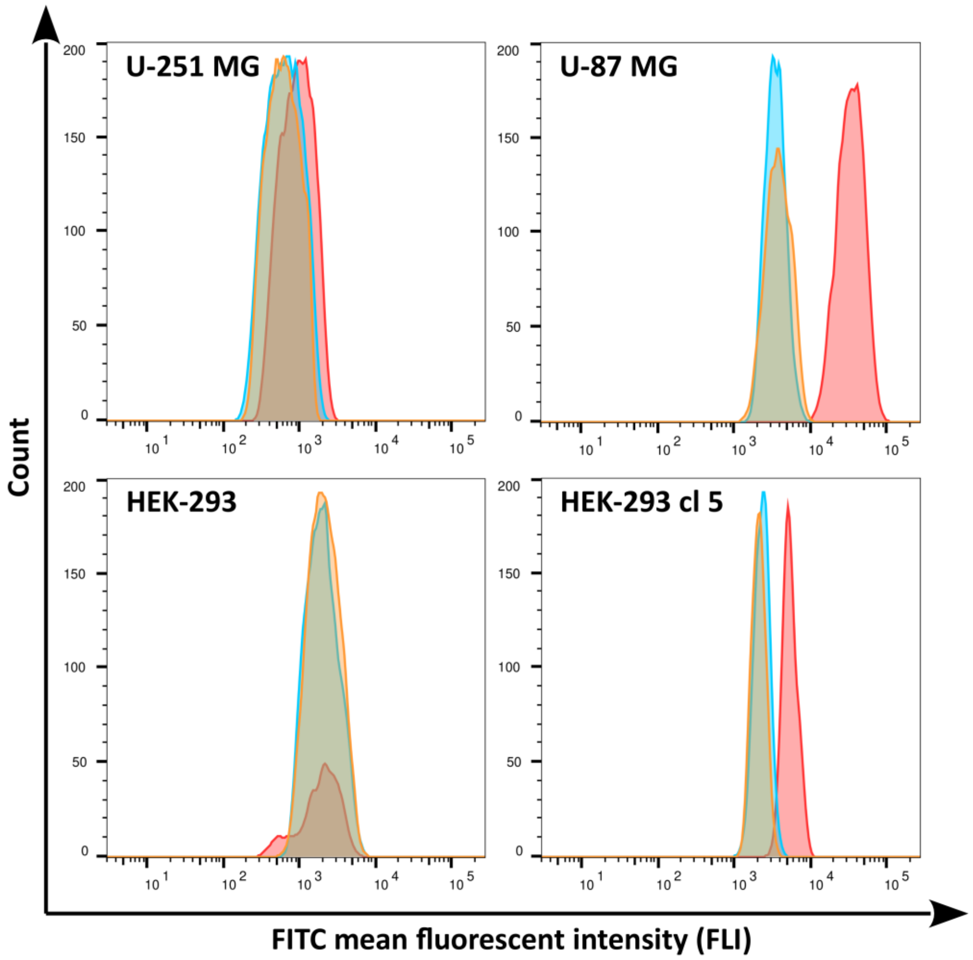
U-251 MG
U-87 MG
HEK-293
HEK-293 cl 5
Count
FITC mean fluorescent intensity (FLI)
200
150
100
50
0
10 1
10 2
10 3
10 4
10 5

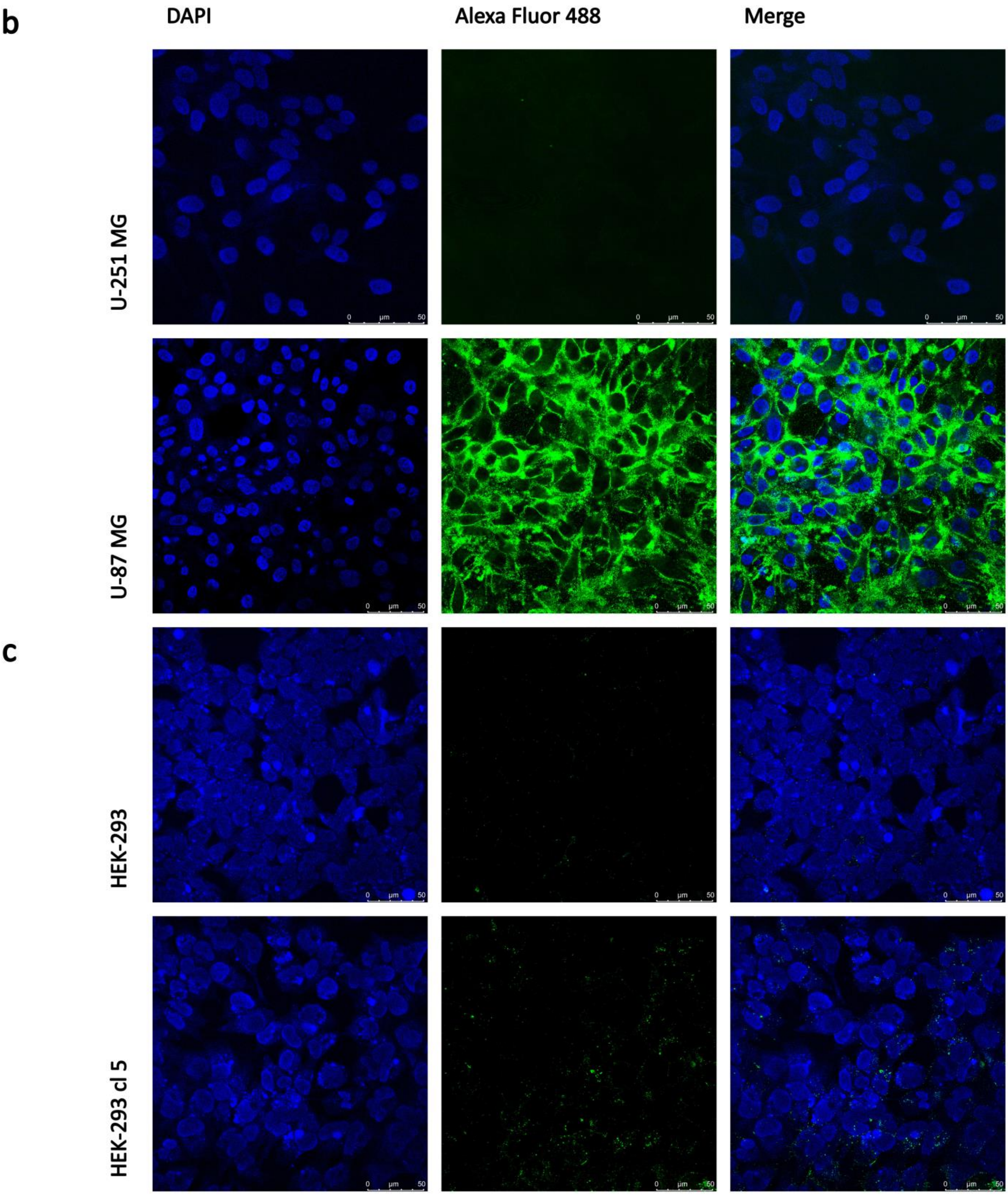

Figure S4. Verification of αVβ3 integrin expression in the cell lines employed. (**a**) Expression was verified with flow cytometry using an antibody that recognizes the αVβ3 heterodimer. Histograms represents fluorescent intensities of cells stained with FITC conjugated anti-αVβ3 integrin antibody (red), isotype IGG control (blue) or unstained cells (orange). (b-c) Confocal microscopic images of the glioblastoma cell line pair (**b**) and the isogenic HEK-293 derivatives (c) detected using the same primary antibody as in (**a**). Acquisition and contrast / brightness settings are identical between the matching pair of cell lines. Ectopic expression of αVβ3 integrin in the HEK-293 clone 5 is lower than that of U-87 MG, but higher than U-251 MG.

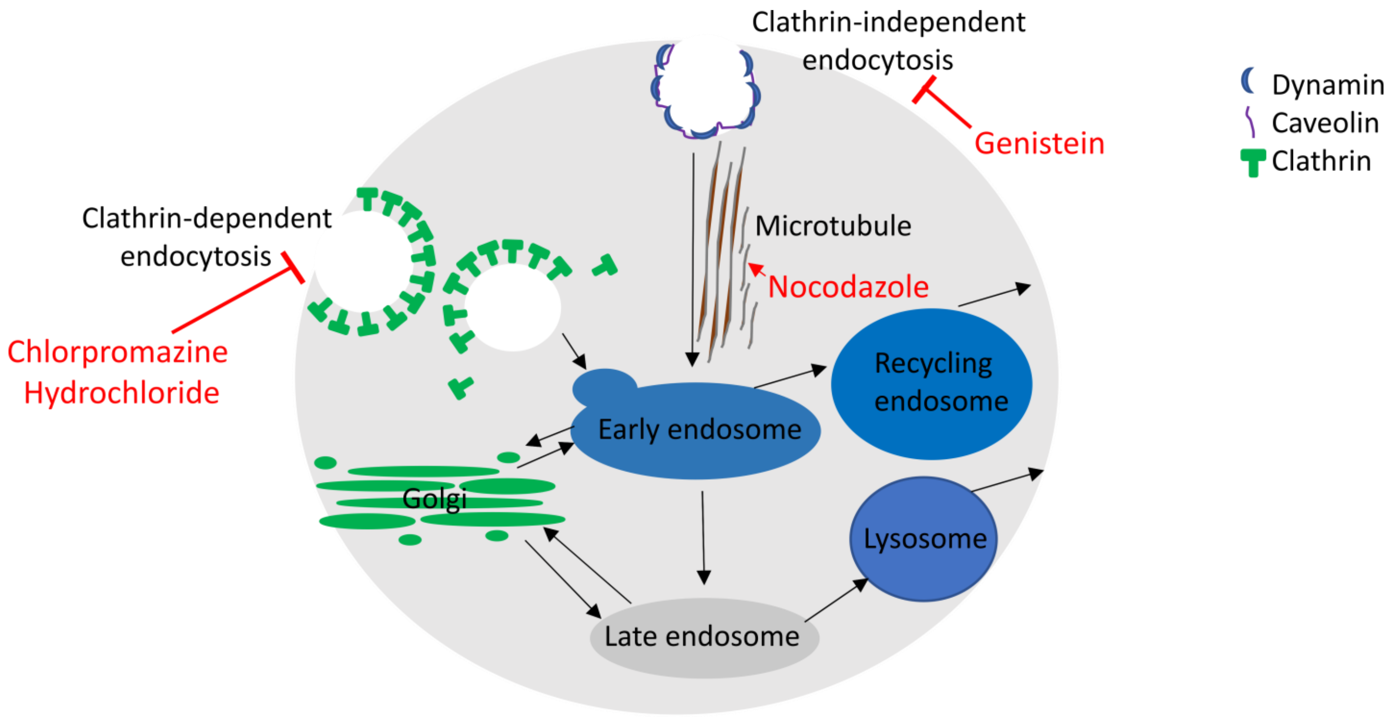

Figure S5. A simplified representation of the clathrin-dependent and -independent mechanisms of endocytosis and their inhibitors.

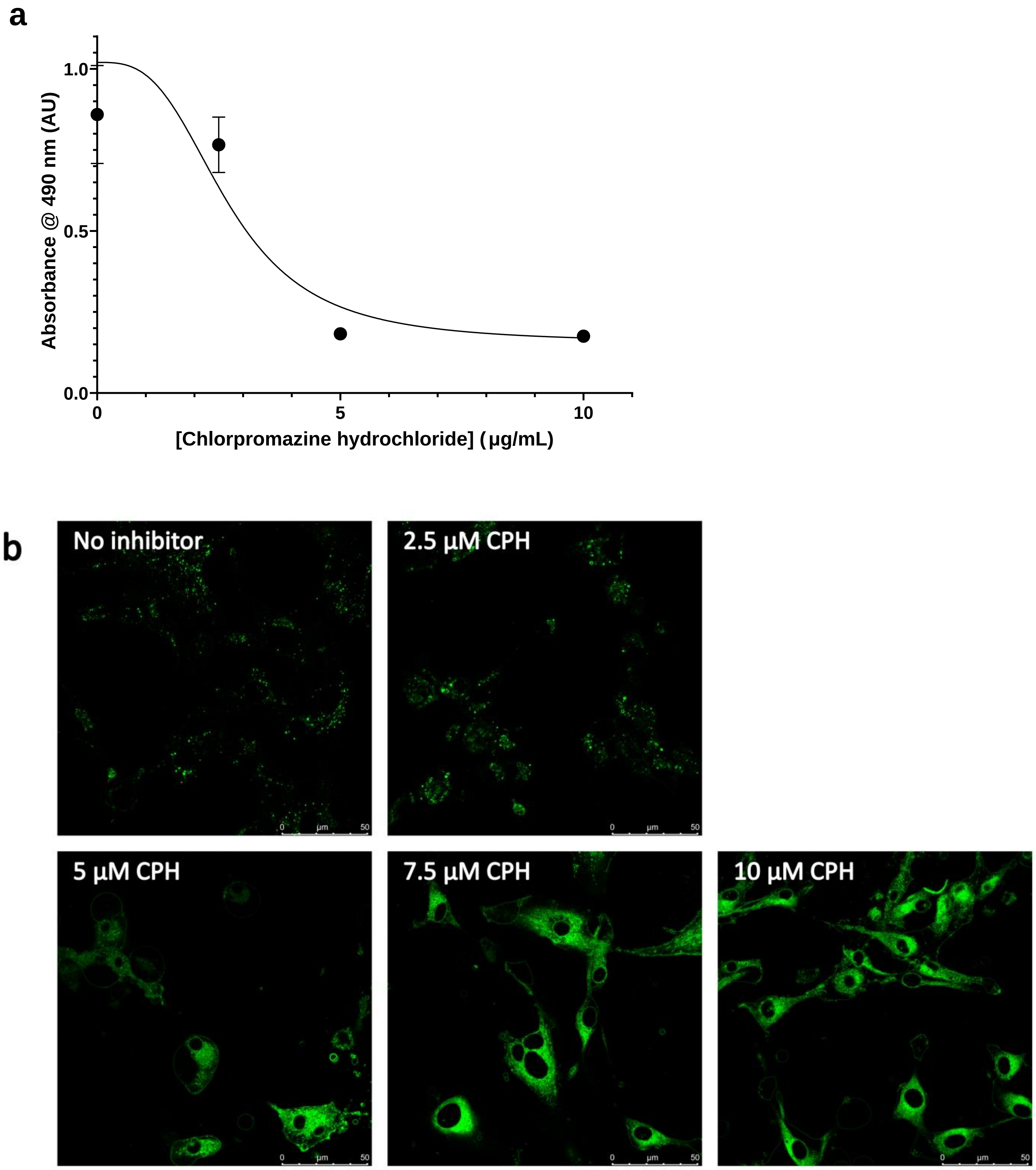

Figure S6. Elevated concentrations of chlorpromazine hydrochloride cause cytotoxicity in U-87 MG cells. (**a**) MTS assay. U-87 MG cells were incubated with the indicated CPH inhibitor concentrations for 2 h then assayed with MTS/PMS as described in the Methods. Line was fitted to the average of the three readings with the Hill equation and is included for information only as the experiment was not designed to establish the EC50 accurately. Error bars represent SD of 3 technical replicates of one experiment. (**b**) Confocal microscopy. U-87 MG cells were incubated with the indicated concentrations of CPH for 30 minutes, then 1 µg/mL LaCer was added. Live cell images were taken 2 h after the addition of LaCer.
